# Sporophytic control of pollen meiotic progression is mediated by tapetum expression of *ABORTED MICROSPORES*

**DOI:** 10.1093/jxb/erac225

**Published:** 2022-05-25

**Authors:** Alison C Tidy, Ivana Ferjentsikova, Gema Vizcay-Barrena, Bing Liu, Wenzhe Yin, James D Higgins, Jie Xu, Dabing Zhang, Danny Geelen, Zoe A Wilson

**Affiliations:** Division of Plant & Crop Sciences, School of Biosciences, University of Nottingham, Sutton Bonington Campus, Loughborough, UK; Division of Plant & Crop Sciences, School of Biosciences, University of Nottingham, Sutton Bonington Campus, Loughborough, UK; Division of Plant & Crop Sciences, School of Biosciences, University of Nottingham, Sutton Bonington Campus, Loughborough, UK; College of Life Sciences, South-Central University for Nationalities, Wuhan, China; Division of Plant & Crop Sciences, School of Biosciences, University of Nottingham, Sutton Bonington Campus, Loughborough, UK; Department of Genetics and Genome Biology, University of Leicester, Leicester, UK; Joint International Research Laboratory of Metabolic & Developmental Sciences, School of Life Sciences and Biotechnology, Shanghai Jiao Tong University, Shanghai, China; Joint International Research Laboratory of Metabolic & Developmental Sciences, School of Life Sciences and Biotechnology, Shanghai Jiao Tong University, Shanghai, China; School of Agriculture, Food and Wine, University of Adelaide, Waite Campus, Urrbrae, South Australia, Australia; Department of Plant Production, Ghent University, geb. A, Gent, Belgium; Division of Plant & Crop Sciences, School of Biosciences, University of Nottingham, Sutton Bonington Campus, Loughborough, UK; Ohio State University, USA

**Keywords:** ABORTED MICROSPORES, AMS, anther, callose, cytokinesis, male sterile, meiosis, pollen development, radial microtubule array, tapetum

## Abstract

Pollen development is dependent on the tapetum, a sporophytic anther cell layer surrounding the microspores that functions in pollen wall formation but is also essential for meiosis-associated development. There is clear evidence of crosstalk and co-regulation between the tapetum and microspores, but how this is achieved is currently not characterized. ABORTED MICROSPORES (AMS), a tapetum transcription factor, is important for pollen wall formation, but also has an undefined role in early pollen development. We conducted a detailed investigation of chromosome behaviour, cytokinesis, radial microtubule array (RMA) organization, and callose formation in the *ams* mutant. Early meiosis initiates normally in *ams*, shows delayed progression after the pachytene stage, and then fails during late meiosis, with disorganized RMA, defective cytokinesis, abnormal callose formation, and microspore degeneration, alongside abnormal tapetum development. Here, we show that selected meiosis-associated genes are directly repressed by AMS, and that AMS is essential for late meiosis progression. Our findings indicate that AMS has a dual function in tapetum–meiocyte crosstalk by playing an important regulatory role during late meiosis, in addition to its previously characterized role in pollen wall formation. AMS is critical for RMA organization, callose deposition, and therefore cytokinesis, and is involved in the crosstalk between the gametophyte and sporophytic tissues, which enables synchronous development of tapetum and microspores.

## Teaser

The tapetum transcription factor ABORTED MICROSPORES is key to tapetum–meiocyte crosstalk by enabling late meiosis progression, cytokinesis, radial microtubule array organization, and callose deposition.

## Introduction

The tapetum is one of the most important cell layers in the anther. It is in direct contact with the developing pollen, plays an uncharacterized regulatory role in meiosis, and is essential for the subsequent biosynthesis and control of pollen wall formation (Zhang and Yang, 2014). Tapetum cell differentiation coincides with anther meiotic development, with the tapetum having a major secretory function in pollen wall formation and pollen maturation by providing enzymes for microspore release from tetrads, sporopollenin biosynthesis, and secretion of pollen wall components (Liu and Fan, 2013; Shi *et al.,* 2015; Wang *et al.,* 2018). Meiosis occurs in Arabidopsis stage 6 anthers, and several meiotic regulators involved in pollen development have been identified (Sanders *et al.,* 1999; Chang *et al.,* 2011; Caryl *et al.,* 2003; Liu and Qu, 2008).

Although the tapetum is critical in supporting pollen development following the completion of meiosis, there has been uncertainty regarding its precise role in meiosis. A number of mutants lacking a differentiated tapetum cell layer, such as *excess microsporocytes1* (*ems1*) and *tapetum determinant1* (*tpd1*), indicate that a functional tapetum is required for the completion of meiosis (Zhao *et al.,* 2002), but the initiation of meiosis in these mutants occurs apparently normally. The control of tapetum development has been shown to be tightly regulated, with rapid turnover of specific proteins and feed-forward and feed-back regulatory loops. These are controlled by key tapetum-expressed transcriptional factors such as DYSFUNCTIONAL TAPETUM1 (DYT1), DEFECTIVE IN TAPETAL DEVELOPMENT (TDF1), ABORTED MICROSPORES (AMS), MALE STERILE 188 (MS188/MYB80/MYB103), and MALE STERILE 1 (MS1), which are part of a regulatory cascade directing pollen development (Fu *et al.,* 2014; Cui *et al.,* 2016; Xiong *et al.,* 2016; Wang *et al.,* 2018). The *ams*, *dyt1*, and *tdf1* mutants are defective in tapetum function around the time of meiosis and therefore AMS, DYT1, and TDF1 are potential key players regulating transcription associated with meiotic stages in male microspores. These mutants show tapetum hypertrophy and microsporocyte degeneration (Sorensen *et al.,* 2003; Zhang *et al.,* 2006), and this tapetum phenotype differs from other tapetum male sterile mutants that are later in the developmental progression such as *ms188/myb80* and *ms1*, which produce single microspores that then degenerate (Yang *et al.,* 2007; Zhang *et al.,* 2007; Xu *et al.,* 2014).

AMS encodes a basic helix–loop–helix (bHLH) protein that is expressed specifically in the tapetum; it shows biphasic protein expression starting at anther stage 5–6 (pre-meiotically), declining and then increasing from the free microspore to bicellular pollen stages (stage 8–11) (Ferguson *et al.,* 2017). *MS188/MYB80* is a direct downstream target of AMS, and both have established roles in sporopollenin formation (Fu *et al.,* 2014; Cui *et al.,* 2016; Xiong *et al.,* 2016); extensive gene expression changes (549 genes) are observed in the *ams* mutant, including direct regulation of 23 genes involved in sporopollenin biosynthesis and secretion (Xu *et al.,* 2010, 2014). This late role of AMS in pollen wall formation is well established, but we have shown that functional AMS protein is also required during early pollen development (Ferguson *et al.,* 2017). Here we have investigated this early role of AMS during meiosis and tetrad formation and have shown that AMS is critical for radial microtubule array (RMA) organization, callose deposition, and cytokinesis to allow correct tetrad formation, alongside its established subsequent role later in pollen wall development.

## Materials and methods

### Plant materials and growth conditions

Arabidopsis Columbia-0 (Col-0) was used as wild-type; the mutants and transgenic lines used were SALK T-DNA line *ams* (Sorensen *et al.,* 2003); ethyl methane sulfonate mutant *tdf1* (Zhu *et al.,* 2008), transposon tagged lines *dyt1-3*, *ms188-3*, and *ms1-8* (Zhu *et al.,* 2011); and inducible line AMSprom:AMS-GR-YFP in Col-0 background (where GR is DEX-binding domain of the rat glucocorticoid receptor; Ferguson *et al.,* 2017). Lines were grown according to Ferguson *et al.* (2017).

### Cytology and microscopy

Fixation and preparation of slides for basic cytology was as described by Higgins *et al.* (2014). Terminal inflorescences were fixed in 3:1 ethanol:glacial acetic acid (EAA) overnight and then stored at −20 °C. Fixed flower buds from a single inflorescence were separated to remove the post-meiosis buds for meiotic analysis, or collected based upon size range for stage analysis. Anthers were carefully isolated prior to enzymatic digestion. Buds were washed twice in 10 mM citrate buffer pH 4.5 at room temperature, then incubated in citrate buffer containing 0.3% w/v cytohelicase (Sigma, cat. no. C1794), 0.3% (w/v) pectolyase (Sigma, cat. no. C8274) and 0.3% (w/v) cellulase (Sigma, cat. no. P5936) for 30 min to 1 h in a humid chamber at 37 °C. Replacing the enzyme mixture with ice-cold citrate buffer stopped the reaction.

Meiotic progression was determined by staining pollen mother cells (PMC) from isolated anthers using 1 µg μl^−1^ 4ʹ,6-diamidino-2-phenylindole (DAPI; Sigma) in Vectashield (Vector Laboratories) anti-fade mounting medium after squashing and UV observation. Over 300 meiotic cells were imaged to follow meiotic progression. Transition electron microscopy (TEM) samples were treated as described by Xu *et al.* (2010) and analysed according to W. Chen *et al.* (2011). Callose staining was performed by releasing meiotic cells in a drop of aniline blue solution (0.1% in 0.033% K_3_PO_4_). Over 200 meiotic cells were observed by callose staining. Semi-thin sections (0.5 µm) were stained with alkalinized 1% toluidine blue.

### 5-Ethynyl-2ʹ-deoxyuridine labelling for meiotic progression

Wild-type and *ams* flowering stems were cut under water and quickly transferred to 1 mM 5-ethynyl-2ʹ-deoxyuridine (EdU) solution for 2 h for uptake by their transpiration stream and incorporation into cells in S-phase. Stems were then removed, ends rinsed, and placed into distilled water for the time course analysis. Whole inflorescences were fixed and prepared for cytology as described previously. Digested anthers were placed in 10 μl 60% (v/v) acetic acid, re-fixed in cold 3:1 EAA fixative, and then the slides were dried. Meiosis progression was detected using Click-IT EdU Alexa Fluor 488 imaging (from step 4.1 of ClickIT kit protocol; Thermo Fisher Scientific). Slides were mounted in Vectashield and observed (488 nm). Three biological replicates were performed, and six digested anthers were analysed per slide. Over 400 meiotic cells were imaged as part of this time course, and the latest stage of development seen per time point was used to mark meiotic progression.

### α-Tubulin immunolocalization

α-Tubulin immunolocalization was performed according to De Storme *et al.* (2012) with minor modifications. Inflorescences were treated with *m*-maleimidobenzoyl *N*-hydrosuccinimide ester (100 mM in 50 mM potassium phosphate buffer and 0.05% Triton X-100, pH 8; 30 min under vacuum) and fixed in 4% paraformaldehyde, then washed in 50 mM potassium phosphate buffer (pH 8) and digested as above for 90 min. After the first digestion, anthers were dissected, squashed, and fixed on a slide by freezing in liquid nitrogen. Released cells on the slide were then immobilized with a thin layer of 1% gelatine, 1% agarose, and 2.5% glucose, and digested again for 90 min at 37 °C. After rinsing with potassium phosphate buffer, immobilized cells were then incubated overnight at room temperature with rat α-tubulin primary antibody (0.3%; clone B-5-1-2; Sigma-Aldrich) in phosphate-buffered saline (PBS) containing 0.1% Triton X-100 and 4.5 g l^−1^ BSA. Cells were rinsed three times with PBS and incubated for 5 h with 0.5% secondary antibody (labelled goat anti-rat) at 37 °C in the dark. After three PBS rinses, 40 µl of DAPI (2 mg ml^−1^) in Vectashield Antifade Mounting Medium (Vector Laboratories) was added to each slide and they were observed using a fluorescence microscope. Over 150 meiotic cells were imaged for spindle and RMA formation.

### Immunolocalization of meiotic proteins

Following the fixation steps meiocytes were squashed and immobilized on slides based on the protocol by Higgins *et al.* (2014). They were digested for 30 min at 37 °C in the digestion medium, and subsequently incubated for 1 h in PBS–1% Triton X-100 at room temperature. After two rinses with PBS–0.1% Triton X-100, slides were incubated overnight at 4 °C in primary antibodies (rabbit anti-ZYP1, rabbit anti-ASYNAPTIC 1 (ASY1), and rabbit anti-Sad1 and UNC84 domain containing 2 (SUN2) (kindly provided by Profs D. E. Evans and K. Graumann, Oxford Brookes University; Armstrong *et al.,* 2002; Higgins *et al.,* 2005) diluted at 1/100 to 1/300 in PBS–1% BSA, then washed in PBS–0.1% Triton X-100 five times for 10 min. After 2 h incubation at 37 °C with the secondary antibodies in PBS–1% BSA, slides were washed in PBS–0.1% Triton X-100 five times for 10 min and mounted in Vectashield antifade medium (Vector Laboratories) with 80 µg ml^−1^ propidium iodide. Over 50 meiotic cells were imaged with the different antibodies.

### Expression analysis

Closed buds from inflorescences of control (Col-0), *ams*, and AMS:AMS-GR-YFP in the wild-type Col-0 background were collected. AMSprom:AMS-GR-YFP transgenic lines and controls were dipped into 25 µM dexamethasone (DEX) + 0.02% Silwet L-77 and left for 24 h before collection. Total RNA was extracted from inflorescences (~100 mg) (RNeasy Plant Kit, Qiagen). First-strand cDNAs were synthesized from 5 µg total RNA using Superscript III reverse transcriptase (Thermo Fisher Scientific) and an oligo (dT) primer (Thermo Fisher Scientific). qRT-PCR analyses were performed using the Light Cycler 480 real-time PCR system (Roche Applied Science), using Brilliant SYBR Green QPCR Master Mix (Fermentas). At least two biological replicates were analysed, and all samples were run in at least two technical replicates. Primers are listed in Supplementary Table S1. Samples were run using two reference genes, *ACTIN* and *PP2A3*, validation of reference genes was performed using the geNorm method (https://genorm.cmgg.be) for DEX addition (Supplementary Table S2), and then samples were normalized using *PP2A3* reference gene expression based on these results. Relative expression was determined compared with wild-type using the $2^{-\Delta C^{t}}$ analysis method. *In situ* hybridizations were conducted in wild-type buds according to Zhu *et al.* (2011).

### Chromatin immunoprecipitation-qPCR analysis

Chromatin immunoprecipitation (ChIP) analysis of AMS–DNA complexes in wild-type was as described by Xu *et al.* (2010), using their polyclonal AMS-specific antibody (generated using a 522 bp *AMS* fragment); 1.5 g of formaldehyde cross-linked Col-0 buds (0.6 to 1.1 mm) was used with the AMS antibody and no antibody control. A small aliquot of sonicated DNA prior to immunoprecipitation was used as an input control. qRT-PCR was performed with ‘input control’ and ‘no antibody control’ samples included in the analysis. All samples were run with at least two biological replicates and at least two technical replicates. Quantification involved normalization of the cycle threshold (*C*_t_) for each sample by subtracting the *C*_t_ of the input control; fold enrichment was calculated by subtracting the *C*_t_ value of the control (no-antibody).

### Electrophoretic mobility shift assay

The recombinant glutathione *S*-transferase (GST)–AMS protein was prepared using pGEX-4T-1 plasmid (GST-AMS-F/R; Supplementary Table S1). Digoxigenin (DIG)-electrophoretic mobility shift assay (EMSA) probes were synthesized by PCR using E-box promoter segments and labelled with DIG-dUTP (Roche Diagnostics). DNA binding reactions were performed according to Wang *et al.* (2002) with minor modifications. Detection of the electrophoretic bands was performed by alkaline phosphatase-conjugated anti-DIG antibody.

### Tapetum cell sorting

Inflorescences from the tapetum specific A9prom:GFP line (Paul *et al.,* 1992) (kindly donated by Prof. R. Scott, University of Bath) and wild-type (Col-0) were collected and pooled from 20 plants (with at least four biological replicates), and plant cell walls were digested (Protoplast solution: 600 mM mannitol, 2 mM MgCl_2_, 0.1% BSA, 2 mM CaCl_2_.2H_2_O, 2 mM MES hydrate, and 10 mM KCl, pH 5.5; with enzymes 1% cellulase R-10, 0.1% pectolyase, 1% hemicellulase, 1.5% pectinase) at 35 °C, shaking at 85 rpm for 1 h to release protoplasts. Remnants of the buds were removed by filtering through a 70 µm sieve. The solution was then centrifuged (6 min, 200 g) and the pellet resuspended in fresh protoplast solution. This was then filtered through 70 µm and then 40 µm sieves, before flow cytometry (fluorescence-activated cell sorting; FACS). Samples were sorted using a Beckman Coulter Astrios EQ Flow cytometer, equipped with a 488 nm laser and 529/28 nm band pass filter for green fluorescent protein (GFP)/yellow fluorescent protein (YFP) fluorescence. Cells were gated by forward and side scatter profile, and doublet excluded by forward scatter height versus area analysis. GFP fluorescence was identified as signal above wild-type (GFP negative—Col-0) control and cells sorted into protoplast solution. Cells were double sorted to achieved high purity by first using an enrich mode followed by a purify sort mode, and this gave a yield of 1000 cells, which was 0.2% of the initial input. FACS-sorted cells then had RNA extracted using Arcturus PicoPure RNA isolation Kit following the manufacturer’s instructions (Thermo Fisher Scientific). cDNA synthesis and qRT-PCR expression analysis were performed as stated earlier.

### Accession Numbers

Arabidopsis Genome Initiative locus identifiers for the genes described are as follows: *AMS* (At2g16910), *ASY1* (At1g67370), *ZYP1* (At1g22260), *DYT1* (At4g21330), *TDF1* (At3g31050), *MS188/MYB80* (At5g56110), *MS1* (At5g22260) *SPO11* (At1g63990), *ATM* (At3g48190), *ATR* (At5g40820), *SHOC1* (At5g52290), *MS5* (At4g20900), *MPS1/PRD2* (At5g57880), *TPR-like* (At1g04770), *T21.H19* (At5g16280), *ML1* (At5g61960), and At1g33420.

## Results

### *ams* mutants show defects in tetrad formation

**Fig. 1.**
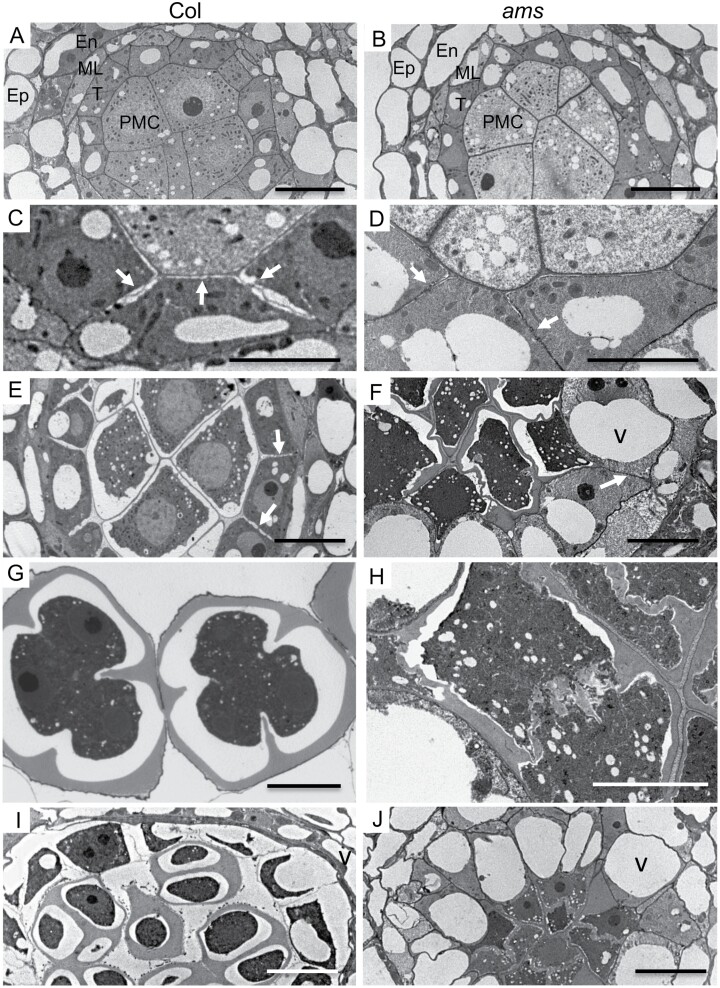
*ams* is male sterile, with abnormal tapetum and tetrads. TEM anther sections of wild-type (WT) Col-0 (A, C, E, G, I) and *ams* mutant (B, D, F, H, J). (A, B) TEM sections of pollen mother cells (PMC) pre-meiosis and tapetum in WT (A) and *ams* (B), showing normal tapetum (T) and PMC, middle layer (ML), endodermis (En), and epidermis (Ep). Meiosis progresses normally in WT (C, E), whereas irregularities occur in the *ams* tapetum with pre-vacuolation (v) mutant tapetal cells (D, F). Connections between tapetum cells and PMC are seen in WT (C, E, arrows), whilst abnormal/compressed connections are seen in the *ams* mutant (D, F, arrows). WT (G) showing callose cell wall deposition on developing meiocytes with cytokinesis occurring to form tetrads, while *ams* (H) has abnormal callose accumulation and separation. Normal tetrads are seen in WT (I) stage (vacuoles: v), whereas abnormal tetrads and highly vacuolated (v) tapetum cells are observed in *ams* (J). Scale bars: 5 μm (C, D, G, H) and 10 μm (the rest).

Abnormalities in *ams* are first detectable during meiosis in TEM sections; in pre-meiosis the PMC and tapetal cells appeared similar in both wild-type and *ams* (Fig. 1A, B; Supplementary Fig. S1A, B). During meiosis in wild-type, the cytoplasm of the tapetum cells became condensed and deeply stained (Fig. 1C; Supplementary Fig. S1C), whereas in *ams* the tapetum cells were swollen with abnormally large vacuoles (Fig. 1D, F; Supplementary Fig. S1D) and only a few lipidic tapetosomes and elaioplasts. The connections between the tapetum cells in the *ams* mutant also appeared impaired, flattened (Fig. 1D, F), and less regular than the connections in the wild-type (Fig. 1C, E). Abnormal cytokinesis occurred in the *ams* meiocytes (Fig. 1H), with irregular tetrad formation and abnormal callose wall (Fig. 1F, H, J; Supplementary Fig. S1D, F), in comparison with the wild-type callose wall and tetrad formation (Fig. 1G).

### Progression of the early meiosis stages of chromosome pairing and synapsis is not disrupted in *ams* anthers

**Fig. 2.**
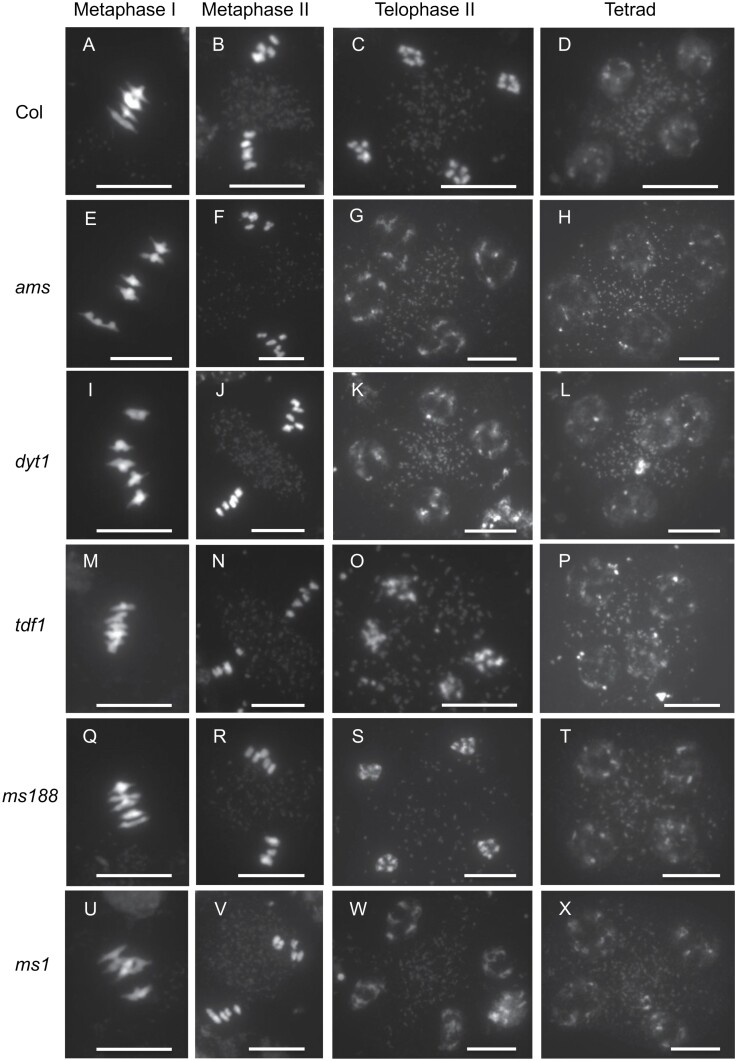
Male sterile mutants have normal early meiosis development. DAPI staining of wild-type (Col) and male sterile mutants *ams*, *dyt1*, *tdf1*, *ms188*, and *ms1* meiocytes during male meiosis. The presence of five bivalents is clear at metaphase I and metaphase II in all lines, with correct separation occurring in metaphase II. During telophase II there is the balanced formation of four sets of five chromosomes with the correct formation of tetrads in all lines observed. Scale bars: 10 μm.

To uncover the mechanism leading to abnormal tetrads in *ams*, we conducted a detailed investigation of chromosome behaviour during meiosis, in *ams* and in other tapetum mutants that are up- and downstream of *AMS*. Meiotic chromosome spreads were prepared using PMCs isolated from the *ams*, *dyt1*, *tdf1*, *ms188*, and *ms1* mutants and compared with wild-type. In wild-type, chromosome dynamics occurred as expected with metaphase I, metaphase II, and telophase II stages having correct chromosome alignment and synapsis, leading to normal tetrad formation (Fig. 2). These early meiosis stages appeared to occur normally in the mutants, but in *ams*, *tdf1*, and *dyt1* abnormal tetrad positioning of nuclei/unbalanced tetrads was frequently seen (26% *ams*, *33% tdf1*, and 60% *dyt1*), whilst the downstream mutants (*ms188* and *ms1*) exhibited normal nucleus positioning within the tetrads (Fig. 2; Supplementary Fig. S2). This suggests that while early meiotic progression occurs normally there are some defects in the final progression to tetrad formation in *ams*, *tdf1*, and *dyt1*.

**Fig. 3.**
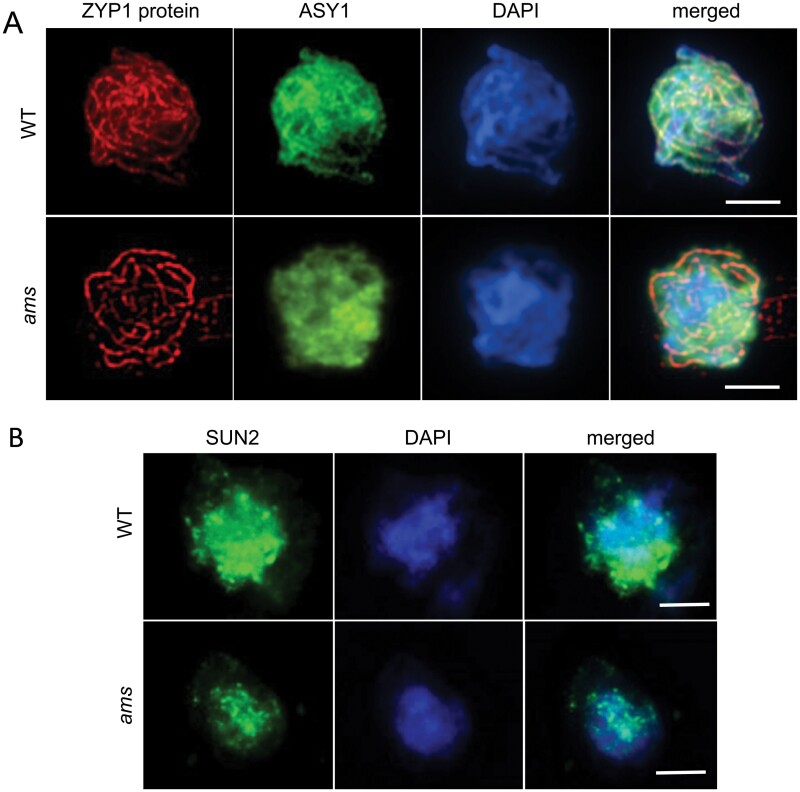
Chromosome pairing, synapsis and nuclear envelope formation appear normal in *ams*. (A) Localization of the synaptonemal complex protein ZYP1 (red) and the axis-associated protein ASY1 (green) at zygotene stage in WT and *ams*. The distribution of both proteins appears normal indicating that chromosome pairing and synapsis occur in the absence of AMS. Chromosomes are counterstained with DAPI (blue). Scale bars: 5 μm. (B) Nucleoporin localization in WT and *ams* pollen mother cells. Similar nuclear envelope expression patterns of AtSUN2 in wild-type and *ams* at telophase II with AtSUN2 (green) antibody, counterstained using DAPI (blue). Strongest SUN2 signal was observed in *ams*. Scale bars: 5 μm.

We further confirmed that early meiotic events were progressing normally by immunolocalization using key meiotic proteins, ASY1 and ZYP1, which are required for normal meiotic progression and crossover formation (Armstrong *et al.,* 2002; Higgins *et al.,* 2005). No differences were observed in the localization of ASY1 and ZYP1 proteins between wild-type and *ams* PMCs (Fig. 3A). Nuclear envelope formation can impact on meiotic progression as shown in the *sun1* and *sun2* double mutants, which have meiotic defects and exhibit a delay in progression of meiosis and an absence of full synapsis (Varas *et al.,* 2015); we therefore analysed immunolocalization of AtSUN2 (SAD2/UNC-48 DOMAIN PROTEIN 2) to determine if nuclear envelope formation was altered in *ams*. The integrity of the nuclear envelope structure appeared similar and regular in both the wild-type and *ams* mutant suggesting the nuclear envelope forms normally in *ams* (Fig. 3B).

### Defects in radial microtubule arrays were observed in the *ams* mutant

**Fig. 4.**
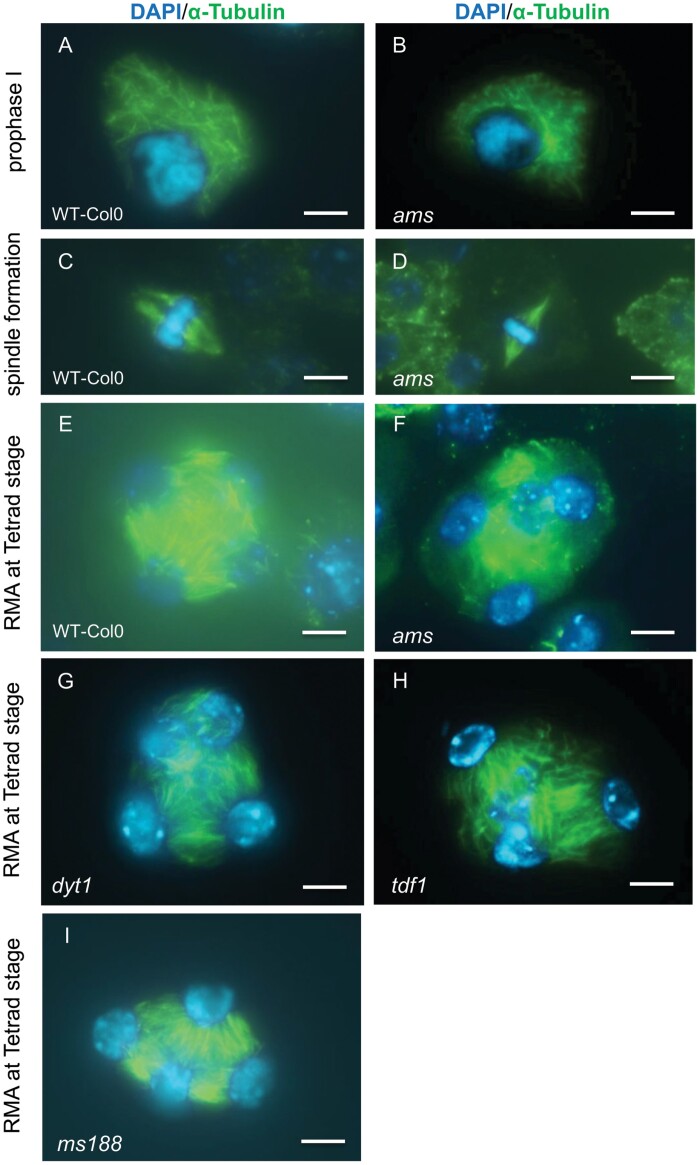
Spindle formation morphology in wild-type (WT, Col 0) and male sterile meiocytes. The spindle was detected by immunostaining with anti-α-tubulin antibody (green) and chromosomes were counterstained with DAPI (blue). Tubulin localization is similar in WT Col-0 and *ams* at prophase I (A, B), with normal spindle morphology during metaphase I in Col-0 (C) and *ams* (D). Radial microtubule arrays (RMA) however are disorganized in *ams* (F) compared with WT (E); this is also observed in the upstream male sterile mutants *dyt1* (G) and *tdf1* (H), but not the downstream mutant *ms188* (I). Nucleus positioning within the tetrad is also unbalanced forming a ‘triad’ like shape in *dyt1*, *tdf1*, and *ams* (G–I). Scale bars: 5 μm.

To understand the timing of meiotic aberrations in *ams* we looked at spindle assembly using immunolocalization of α-tubulin during both early and late stages of meiosis. During early stages around prophase I, wild-type and *ams* meiocytes had very similar perinuclear microtubule arrangements (Fig. 4A, B); no abnormalities were also observed later during metaphase I with *ams* also showing normal spindle morphology (Fig. 4C, D). These data suggest that early meiosis is not affected in the *ams* mutant. However, normal single microspores are not formed in *ams*, and it is evident that the tetrad stage is unstable with defects observed during cytokinesis (Fig 1F). Meiosis cytokinesis depends on the formation of RMAs through interaction of actin filaments and microtubules with the microtubule organizing centres on the surface of telophase II nuclei (De Storme and Geelen, (2013). During the late tetrad stage, defects in RMAs were analysed using an α-tubulin marker, with disorganization of RMA observed in *ams* (Fig. 4F). This disorganization was also seen in the *AMS* upstream mutants, *dyt1* and *tdf1* (Fig. 4G, H), but not in the downstream *ms188* mutant (Fig. 4I). Disorganization of RMAs is linked to abnormal nuclei positioning within the tetrad, resulting in the formation of unbalanced tetrads, such as the ‘triad’ distribution of the four nuclei (meiotic restitution), due to disorganization of microtubules forming between the nuclei. This phenotype was observed in the *ams*, *dyt1*, and *tdf1* mutants, which all fail to express *AMS*, but not in the later mutant *ms188* (Fig. 4), this suggests that functional *AMS* may play a key role in the control of cytokinesis and RMA organization, which allows normal tetrad formation.

### Callose cell wall is abnormal in *ams* mutant tetrads

**Fig. 5.**
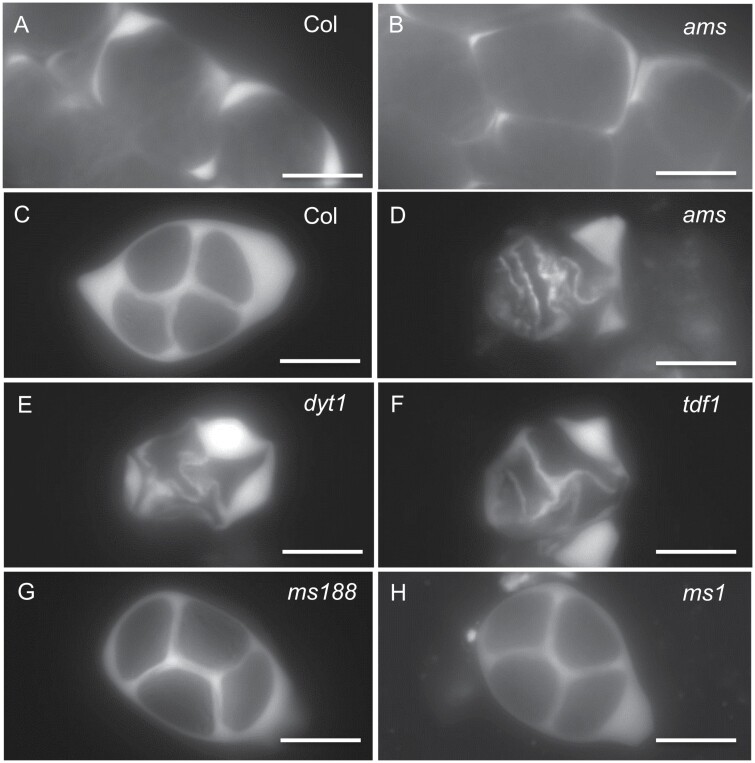
Callose staining of male meiocytes of tapetum defective mutants. Callose staining of wild-type (WT) Col-0 (A) and *ams* (B) tetrads during meiosis initially showed similar callose production, suggesting normal initiation of callose biosynthesis. Callose deposition, forming thick walls surrounding the tetrads, was subsequently seen in WT (C), whereas this was abnormal and disorganized in *ams* tetrads (D), *dyt1* (E), and *tdf1* mutants (F), and the later stage tapetum mutants, *ms188* (G) and *ms1* (H), showed normal callose deposition and thick, ordered callose layers surrounding the tetrads. Scale bars: 10 µm.

Disorganization of the RMA can affect the microspore cell wall as it mediates cell plate formation, and therefore aniline blue staining and analysis of the developing callose cell wall surrounding the tetrads was conducted. Callose wall production was initiated normally in *ams* (Fig. 5A, B), but callose staining was weaker than observed in wild-type (Fig. 5B). *GLUCAN SYNTHASE LIKE 1* (*GSL1*), an essential callose synthase in pollen development (Enns *et al.,* 2005), showed slightly reduced expression at the meiotic stage in the *ams* mutant (FlowerNet: www.cpib.ac.uk/anther; Pearce *et al.,* 2015), which may be associated with the reduced callose staining. There were also defects observed in subsequent callose deposition and cell wall organization in *ams* (Figs 1H, 5D). This disorganization of the callose cell wall was also observed in the upstream male sterile mutants, *dyt1* and *tdf1* (Fig. 5E, F), but not in downstream *ms188* and *ms1* mutants (Fig. 5G, H). This may be a direct consequence of the disorganization of the RMA, or that AMS itself plays an important role in callose cell wall deposition as previously proposed (Xu *et al.,* 2010, 2014). Alternatively, this could also be a consequence of the tapetum hypertrophy that is observed in *ams* and the upstream mutants *tdf1* and *dyt1.* As well as disorganization of callose cell wall in these three mutants, there is also an associated compaction: in the wild-type tetrads are well-separated whereas they are observed adjacent to each other in *ams*, *dyt1*, and *tdf1* mutants (Supplementary Fig. S3). This compaction could be due to tapetum hypertrophy, and/or may reflect impaired callose deposition/breakdown, since AMS has been previously reported to directly regulate *Anther-specific protein 6* (*A6*) whose gene product has been proposed to act in callose breakdown (Xu *et al.,* 2010, 2014).

### Time-course analysis of meiotic progression revealed a significant delay in *ams* mutant

**Fig. 6.**
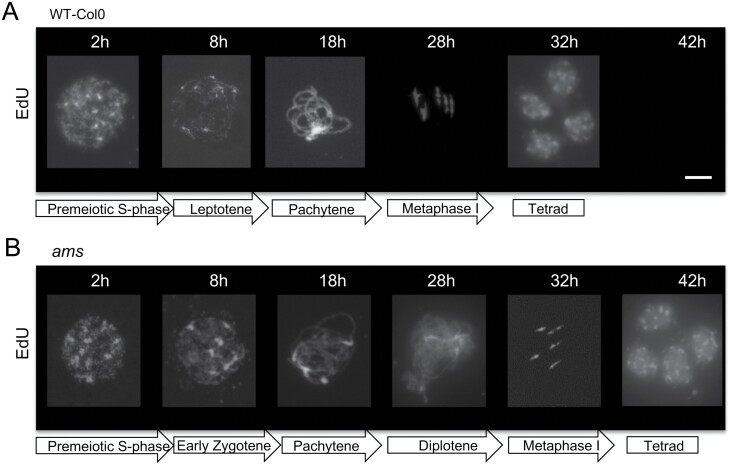
Meiotic progression over time in *ams* compared with wild-type. Detection of EdU labelling in pollen mother cells across a time course of sampled cells (2, 8, 18, 28, 32, and 42 h). White, EdU Alexa Fluor 488. (A) Wild-type (WT) Col-0 showing normal meiotic progression, with tetrads observed after 32 h. (B) *ams* showed delayed progression through meiosis after pachytene stage, with occasional tetrads observed only after 42 h. Scale bar: 10 μm.

The *ams* mutant exhibits a more severe impact on fertility and a complete male sterile phenotype compared with other RMA and callose mutants, suggesting that AMS has additional impacts on pollen development. The duration of meiosis was therefore observed to see if mistiming of pollen development, with associated abnormal tapetum development, was occurring. A detailed analysis of the temporal progression of meiosis in the *ams* mutant was conducted using EdU labelling of meiocytes, focusing on the timing of meiotic phases compared with wild-type. EdU was successfully incorporated into newly synthesized DNA (pre-meiotic S-phase) in both wild-type and *ams* meiocytes in a 2 h window of EdU labelling. However, while wild-type progressed through the subsequent stages as expected, the EdU labelled time course of *ams* meiocytes showed delayed progression after the pachytene stage and retarded entry into the later meiotic stages. Wild-type meiocytes progressed quickly to metaphase I, and tetrads could be detected by 32 h from the point of EdU labelling (Fig. 6A). The *ams* mutant, however, had prolonged progression through pachytene/diplotene/metaphase I; normal tetrads were only occasionally observed in the mutant, but this was not until after 42 h (Fig. 6B) rather than the normal 32 h seen in wild-type. This suggests that while early meiosis occurs normally the progression itself is delayed through the stages in *ams*, which may contribute to failure of microspore development by misaligning tapetum and microspore development. This proposed delay in meiotic progression was also indicated by observations of bud sizes; larger buds were seen in *ams* from PMC onwards compared with wild-type, with meiosis occurring in smaller buds in wild-type than in the corresponding stage in *ams* (Supplementary Fig. S4). Increased bud size has been previously linked to prolonged meiosis in meiotic mutants (Z. Chen *et al.,* 2011), and this further supports the hypothesis of delayed meiotic progression in *ams*.

### AMS binds the promoter regions of six genes associated with meiosis

**Fig. 7.**
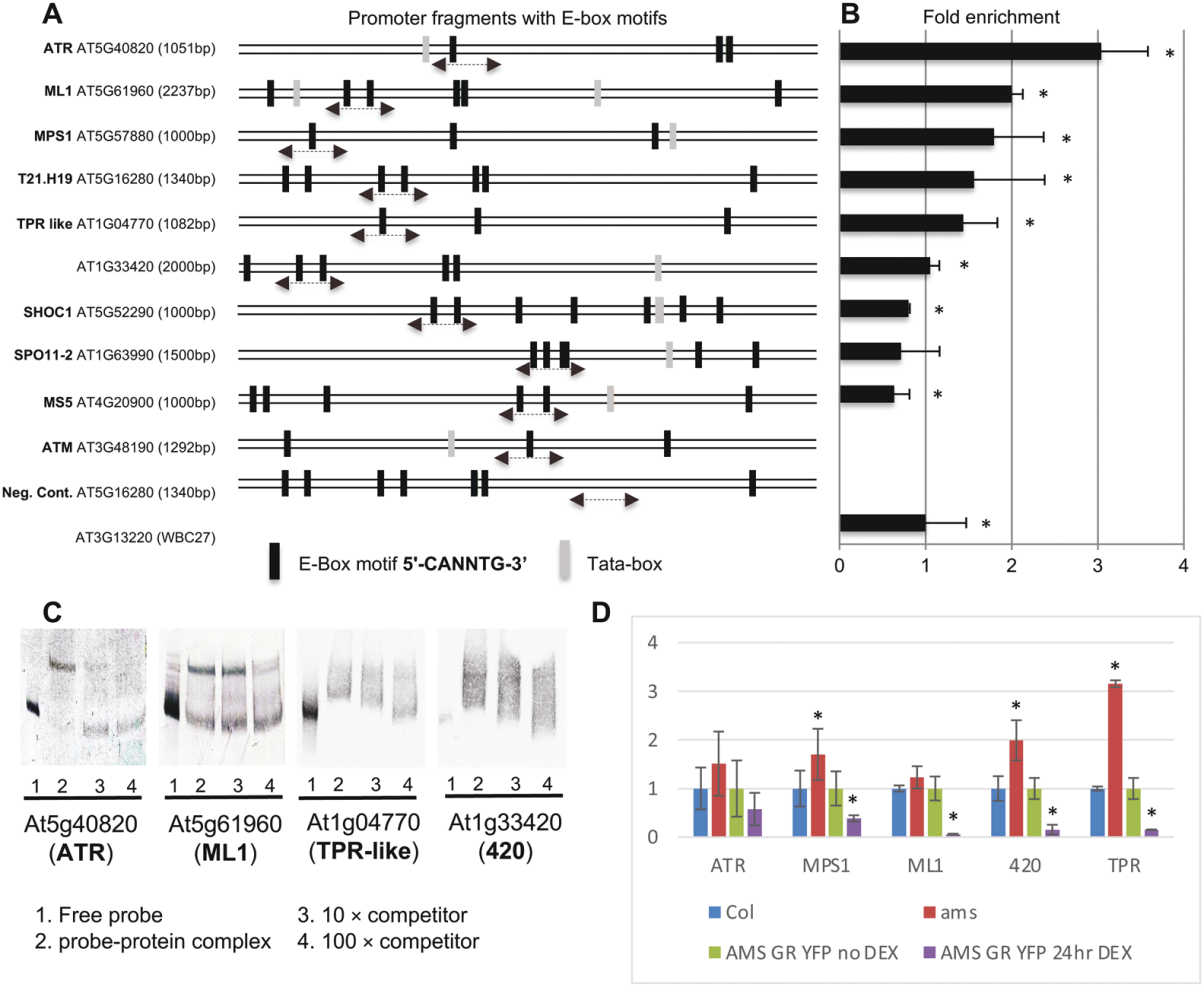
AMS binds directly to the promoters of selected meiotic-associated genes to regulate their expression. (A) ChIP–qPCR analysis of the enrichment of AMS regulatory targets compared with WBC27 positive control. Predicted E-boxes in the promoter region represented by dark vertical lines and promoter regions analysed by ChIP–qPCR and EMSA represented by dotted line and arrows. (B) Fold enrichment represents the fold change in +Ab (antibody) compared with −Ab samples, normalized to WBC27 fold change. qPCR data were gathered from three biological and two technical replicates. *Significant changes based on Student’s *t*-test, *P*<0.05. Error bars represent SD. (C) EMSA using digoxigenin-labelled probes without AMS protein or unlabelled probes (lane 1); lanes 2–4 show AMS protein and DIG-labelled probe with increasing amount of competitor DNA (10× and 100×, respectively). Gel retardation indicates the binding of the AMS to promoters of the target genes. (D) Relative expression values based on qRT-PCR analysis measured in whole inflorescence in wild-type (Col-0), *ams*, and AMSprom:AMS-GR-YFP in WT Col-0 background, showing up-regulation in *ams* mutant and down-regulation in the AMSprom:AMS-GR-YFP line 24 h after AMS induction by DEX. Expression was normalized to wild-type for *ams* and to AMS-GR-YFP without DEX for AMS-GR-YFP 24 h after DEX. *Significant changes based on Student’s *t*-test, *P*<0.05. Error bars represent SE.

Meiotic-associated genes *SPO11* (At1g63990), *ATM* (At3g48190), *ATR* (At5g40820), *SHOC1* (At5g52290), *MS5* (At4g20900), *MPS1/PRD2* (At5g57880), *TPR-like* (At1g04770), *T21.H19* (At5g16280), *ML1* (At5g61960), and At1g33420 (Table 1) showed altered expression in *ams* (Xu *et al,.* 2010) and interaction with AMS protein in preliminary ChIP-Seq studies. This suggests that AMS may be involved in direct regulation of these genes. The 1–2 kb promoter/upstream sequences of these putative meiosis-associated target genes were examined for motifs by the TRANSFAC (Transcription Factor Binding Sites) tool (www.biobase-international.com) for presence of AMS binding E-box elements. At least three E-box binding motifs were observed for each target (Fig. 7A), and no other enrichments of common motifs were identified. These regions were used to generate 150–250 bp PCR fragments to test for AMS binding to the target promotor regions by ChIP–PCR analysis.

**Table 1.** Putative AMS target genes associated with pollen mother cell meiosis.

AMS putative target gene	Gene name	Known function in Arabidopsis	Associated references
At1g63990	*SPO11-2*	Endonuclease responsible for the induction of DNA double strand breaks during meiosis	Stacey *et al.* (2006); Hartung *et al.* (2007)
At5g52290	*SHOC1*	Required for class I cross-overs during meiosis	Macaisne *et al.* (2011)
At5g57880	*MPS1/PRD2*	Involved in DNA double strand break formation and spindle organization in meiocytes; transcript expression reached its highest level in male meiocytes	de Muyt *et al.* (2009); Jiang *et al.* (2009)
At4g20900	*MS5*	Abnormalities after meiosis II in *ms5*, possibly due to disturbances in meiosis I or in proteins of the synaptonemal complex	Glover *et al.* (1998)
At5g61960	*ML1*	Meiotic abnormalities: pairing defects, fragmentation, and clumping of chromosomes	Kaur *et al.* (2006)
At1g33420	*—*	RING/FYVE/PHD zinc finger superfamily protein; likely involved in transcription	—
At1g04770	*TPR-like* *SID2*	48% identity with MS5 family protein	Blatch and Lassle (1999)
At3g48190	*ATM*	Signal transducer in DNA damage repair machinery, signals the existence of DNA double-strand breaks	Garcia *et al.* (2003)
At5g40820	*ATR*	Signals the presence of DNA single-stranded breaks, mostly at stalled replication forks	Culligan *et al.* (2004)
At5g16280	*T21.H19*	Tetratricopeptide repeat (TPR)-like superfamily protein; 96.5% identity with *Arabidopsis lyrata* involved in protein trafficking	—

Three independent ChIP experiments were conducted to test for enrichment of the target fragments in DNA immunoprecipitated using an AMS antibody (previously generated for ChIP by Xu *et al.*, 2010). The enrichment was calibrated to a positive control of *WBC27* (Xu *et al.*, 2010). All 10 genes tested showed enrichment by ChIP–PCR analysis and therefore may be direct AMS targets, but six of these genes (*ATR*, *MPS1/PRD2*, *TPR-like*, *T21.H19*, *ML1*, and *At1g33420*) showed significant enrichment equivalent or greater than that observed for the positive control, *WBC27* (Fig. 7B). EMSA was subsequently employed to confirm AMS binding to the promoters of four of these putative targets. Purified AMS protein was used to probe E-box rich promoter fragments of the target genes. Retardation was seen with all the genes tested (Fig. 7C), indicating positive protein–DNA interactions. To demonstrate binding specificity, a 10-fold and 100-fold excess of unlabelled probe was added to the EMSA reaction as competitor. The specific complex was greatly reduced by the addition of the unlabelled competitors, particularly for *ATR*, *TPR-like*, and *ML1*, thus confirming the specificity of interactions between AMS protein and the E-box enriched promoter fragments.

### AMS acts as a regulator of meiosis putative AMS targets

Five of the meiosis-associated putative AMS target genes were further analysed for their expression profiles by qRT-PCR in unopened buds. All of these genes showed up-regulated expression in the *ams* mutant compared with wild-type, which was normalized to 1 to aid comparison (Fig. 7D), suggesting that AMS may negatively regulate the expression of these putative targets. The effect of functional AMS induction was then tested using a DEX-inducible AMS construct (AMSprom:AMS-GR-YFP from Ferguson *et al.*, 2017), which 24 h after DEX treatment resulted in AMS protein localized to the nucleus and a reduction in the expression of all the putative targets when compared with AMS–GR–YFP prior to DEX treatment (normalized to 1 to aid comparison), with TPR-like showing the strongest association (Fig. 7D).

AMS has been detected in the tapetum with no meiocyte expression observed (Ferguson *et al.,* 2017), and therefore expression analysis was performed on the meiosis-associated genes to determine if these target genes were also present in the tapetum. Isolated tapetum cells, expressing the tapetum-specific A9prom:GFP transgene (Paul *et al.,* 1992; kindly provided by Prof. R. Scott), were enzymatically separated and subject to FACS based on the GFP marker, and then used for qRT-PCR. Enrichment of the selected meiotic-associated AMS target genes was seen within the tapetum enriched samples (Supplementary Fig. S5A). In these tapetum cells all of the meiotic genes tested, except for *MPS1*, were present, but early meiotic genes such as *ML1*, *TPR-like*, and *At1g33420* showed higher enrichment. This may be a reflection of the fact the early meiotic cells were easier to release during enzyme digestion for cell sorting, since this was also seen in the strong enrichment of *DYT1* (early meiosis) compared with *AMS* (late meiosis). Tapetum localization was further confirmed for one of these genes, *ML1*, through *in situ* hybridization (Supplementary Fig. S5B–D). This co-localization of expression further supports the potential direct interaction between AMS and *ATR*, *TPR-like*, *T21.H19*, *ML1*, and *At1g33420* within the tapetum. This suggests that AMS repression of these targets may be needed for progression of the final stages of meiosis, but that this interaction may occur within the tapetum, but the subsequent impact of this is manifested in the gametophyte.

## Discussion

AMS is a bHLH transcription factor that acts as a key player in tapetum development via the direct regulation of many genes (Ferguson *et al.*, 2017) and thus plays an important role in viable pollen formation, with major impacts on late meiotic events as well as its well-characterized later role during pollen wall formation.

### Callose wall formation is impaired in *ams* during meiosis

In the *ams* mutant early meiosis initiates normally (Figs 2, 3), but there is a failure during late meiosis with abnormalities such as callose wall formation observed (Fig. 5), which ultimately leads to microspore degeneration (Fig. 1). Callose wall deposition is important for establishing the matrix for pollen wall formation and the generation of viable pollen. This change in callose wall formation may be a direct effect of the lack of *AMS* as AMS itself plays an important role in callose cell wall deposition as previously proposed (Xu *et al.,* 2010, 2014). Alternatively, tapetum hypertrophy occurs in the *dyt1*, *tdf1*, and *ams* mutants and appears to result in the tapetum filling the locule and squeezing the meiocytes; this could potentially impact the callose wall formation/dissolution, especially as the meiocytes appear close together in these three mutants, rather than well separated as in the wild-type (Supplementary Fig. S3). Mutants in callose deposition at the developing cell plate such *gsl1/gsl5* double mutants, have no callose wall in-between the developing tetrads resulting in problems in tetrad dissociation, failed cytokinesis and pollen abortion; nevertheless, they are able to produce some abnormal larger pollen with multiple nuclei (Enns *et al.,* 2005). However, the phenotype observed in *ams* is more severe, with no single microspores formed and full sterility, suggesting that AMS is playing an additional role(s) in late meiosis development, leading to failure of cytokinesis and single microspore formation. This is also evident from previous rescue experiments using a DEX-inducible AMS construct, which required multiple DEX treatments to ensure that functional AMS was present at multiple stages to facilitate viable pollen formation (Ferguson *et al.*, 2017). Along with callose, exine deposition is also critical for ultimate pollen viability, with callose and exine mutant phenotypes leading to pollen degeneration (Xu *et al.,* 2014). AMS has a well-established role in exine formation, which may explain why there is ultimately pollen degeneration in this mutant. However, here we have shown it also has an earlier role, which is associated with late meiotic progression, causing abnormalities in intersporal callose deposition during cytokinesis, RMA formation, and cytokinesis.

### AMS plays a role in late meiotic progression through RMA formation and cytokinesis

We have found that early meiosis initiates normally in *ams*, but exhibits delayed progression from the pachytene stage (Fig. 6), and then there is a failure during late meiosis with defects in RMA, cytokinesis, and intersporal callose wall formation (Fig. 4). Mutants such as *tes/stud/Atnack2*, *mpk4*, and *aesp* have problems with disorganized RMA and therefore loss of callose deposition; nevertheless, they can still form monad pollen with multiple nuclei despite disorganized RMA and loss of intersporal callose deposition (Yang *et al.,* 2003; Zeng *et al.,* 2011). The *ams* mutant, however, has a more severe phenotype with meiocytes that are unable to progress past cytokinesis, compared with other mutants involved in RMA formation. Cold stress has also been shown to impact on RMA organization, resulting in diploid and polyploid pollen. De Storme *et al.* (2012) and Spielman *et al.* (1997) have showed that a kinesin mutant with failed cytokinesis led to monad formation, forming tetraploid, or multi-sperm pollen. Defects in RMA do not typically result in inviable pollen, and therefore the observed microspore degeneration in *ams* is unlikely to be the result of the callose/RMA/cytokinesis defect alone. The RMA/triad phenotype of *ams* is similar to a hypomorphic *MPS1/PRD2* mutant (Walker *et al.,* 2018), and therefore affecting the expression of these genes may explain part of the *ams* meiotic phenotype.

### AMS directly represses meiosis-associated genes to mediate crosstalk between the tapetum and meiocytes

**Fig. 8.**
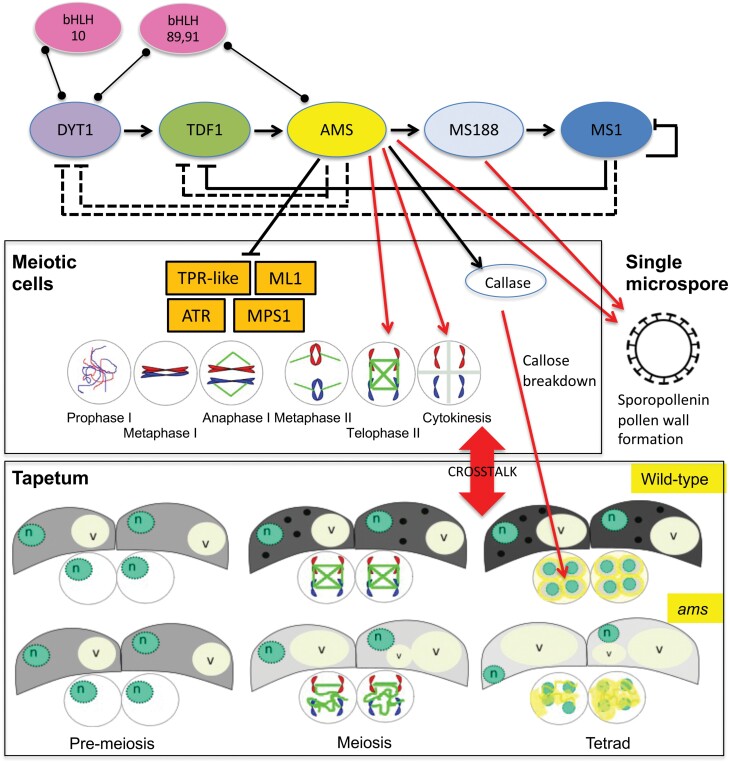
Proposed regulatory network for AMS. Tapetum regulatory pathway based on network published in Ferguson *et al.* (2017). AMS is directly regulated by DYT1 through TDF1, and itself directly regulates MS188, which regulates MS1. AMS has a published role in sporopollenin wall formation alongside MS188. We propose that it is the loss of AMS in the early tapetum mutants that cause the phenotypes observed in *ams*, *tdf1*, and *dyt1* mutants, as *ms188* and *ms1* develop normally. We have shown a novel role for AMS in the correct regulation of RMA localization (green lines in meiocytes) during telophase II, for correct cytokinesis, callose wall deposition (yellow material in meiocyte) to produce a functional tetrad. AMS is also important for fully functional tapetum cells that have a high energy requirement during meiosis (darker background colour represents this, and black circles indicate lipidic tapetosomes and elaioplasts) to provide for the developing pollen. We have shown that AMS appears to directly negatively regulate at least four meiosis-associated genes, possibly through interaction in the tapetum, and propose that AMS has a role in the final stages of meiosis through their regulation. n, nucleus; v, vacuole. Arrows: regulation; lines ending with a line: repression; lines ending with circle: protein interactions; dashed lines indicate a minor role in regulation of network (as predicted by modelling; Ferguson *et al.* 2017); red lines indicate a major role in regulation.

We have shown that selected meiosis-associated genes are directly repressed by AMS (Fig. 7) and suggest that this may be essential for late-stage meiosis progression (Fig. 8). AMS is a tapetum-expressed protein (Ferguson *et al.*, 2017), whereas meiosis occurs within the developing meiocytes; however, we have shown by qRT-PCR of FACS-sorted tapetum cells and *in situ* hybridization, that these meiosis-associated genes are also expressed in the tapetum. Recent work (Li *et al.,* 2017) using laser microdissection also shows expression of *ML1*, *MPS1/PRD2*, and *At1g33420* in stage 6–7 tapetum cells. *ML1* has also previously been shown to be expressed in tapetum cells and meiocytes (Kaur *et al.,* 2006). AMS and the target genes are therefore all expressed within the tapetum and thus AMS may directly interact with the promoters of these genes in the tapetum to change their expression to enable meiotic progression and meiocyte development. AMS is known to be a positive regulator of expression, and therefore repression by AMS may be caused by the binding of AMS to the E-boxes on the target promoters and blocking of access to other transcriptional regulators, thus causing their down-regulation. We show a clear reduction of these meiosis-associated gene transcripts as a consequence of AMS expression, which suggests that their tapetum expression needs to be halted for meiosis to progress and that AMS is coordinating this regulation. This may be due to the movement of RNAs or proteins from the tapetum to the PMCs, since it is indicated that such actions occur as part of the crosstalk between the tapetum and meiocytes (Lei and Liu, 2019), although the factors involved in this are unknown. The cellulose in the tapetum walls is lost prior to meiosis (Matsuo *et al.,* 2013), thus potentially favouring the movement of materials between the tapetum, anther locule, and meiocytes at this stage.

Recently it has been shown that gene targeting siRNAs are produced in tapetal cells and transported into meiocytes possibly through plasmodesmata that connect these cells during early meiosis (Long *et al.,* 2021). Therefore, an alternative hypothesis is that AMS may play a role in activating RNA-mediated gene regulation, such as large intergenic non-coding RNAs (lincRNAs). Recent work (Ono *et al.,* 2018) has indicated that EAT1, a rice tapetal bHLH transcription factor, is able to activate lincRNAs, which are possible mobile signals between the tapetum and reproductive cells and may facilitate negative gene regulation. EAT1 has been shown to have a bimodal expression similar to AMS and also to cause delayed and asynchronous male meiosis in regard to spike size (Ono *et al.,* 2018). We observed this delay and asynchronous meiosis in the *ams* mutant, with increased bud sizes seen in *ams* compared with the corresponding meiosis stages in wild-type buds (Supplementary Fig. S4). The negative regulation of meiotic genes to allow tight control of meiosis and tapetal development may be a way to synchronize the development of these two cell types.

Correct and timely tapetal development has been shown to be very important for the establishment of microspores, and early tapetum mutants such as *dyt1* and *tdf1* also present similar phenotypes to *ams* with RMA disorganization, failed cytokinesis, and abnormal callose wall formation, suggesting that loss of AMS in the upstream transcription factor mutants *dyt1* and *tdf1* may be the principal cause of these phenotypes (Fig. 8). This is supported by the phenotypes of the male sterile mutants downstream of *ams* that do not exhibit these defects, with normal RMA organization and callose deposition seen in *ms188* and *ms1* mutants. All three of these early mutants (*dyt1*, *tdf1*, and *ams*) lack a normal tapetum during this key stage (Zhang *et al.*, 2006; Zhu *et al.*, 2008), indicating that a functional tapetum is critical for completion of meiosis. This suggests that tapetum signals regulating the final progression of meiosis are absent in *ams*, and therefore the *dyt* and *tdf1* mutants. TEM sections of *ams* tapetum cells indicate that they are highly vacuolated and expanded; this occurs significantly earlier than wild-type, with vacuolation from meiosis onwards and a lack of functional tapetum cells, which are important for providing callose synthase, callase, cellulose and possibly energy in the form of sugars for microspore development (Fig. 1C–J). We have observed that the *ams* mutant tapetum has very low amounts of lipidic tapetosomes and elaioplasts, which may impair its ability to provide the necessary building blocks for the early cellulose wall production. The tapetum is grossly enlarged (hypertrophy) at this stage in the *dyt1*, *tdf1*, and *ams* mutants, appearing to fill the locule and squeeze the meiocytes, which may also impact on normal RMA formation, callose deposition, and cytokinesis within the developing meiocytes. Tapetum development does not appear synchronized with the adjacent tetrad formation in *ams* compared with wild-type, suggesting that tapetum development is important for fulfilling the requirements of the meiocytes, which may therefore explain why *ams* exhibits delayed meiotic progression and incomplete pollen wall production. In the future it would be interesting to try to distinguish between the impact of the loss of *ams* and tapetum hypertrophy with its effect on callose formation, RMAs, cytokinesis, tetrad formation, and meiotic progression, for example by testing the impact of DEX-induced AMS expression during early microspore development/meiosis in the *ams*, *dyt1*, and *tdf* mutants, as well as looking at hypertrophic tapetum mutants that are not directly linked to reduction in *ams* expression.

### Conclusions

In summary, we have shown that meiosis initiates and progresses normally in the *ams* mutant until the pachytene stage, where the progression is delayed, with problems apparent at late meiosis stages, with delayed completion of meiosis, disorganized RMA, defective cytokinesis, followed by tetrad collapse and degeneration. This and the subsequent failure in callose and exine development may be the principal causes of the failure of pollen development in *ams*. We have shown that selected meiosis-associated genes are directly repressed by AMS and that this is likely to occur in the tapetum, but that this down-regulation impacts on the meiocytes and is essential for late-stage meiotic progression (Fig. 8). AMS is critical for cytokinesis and RMA organization to allow correct tetrad formation, alongside its established role later in pollen wall development. The work presented here explains the function of our previously reported early peak of AMS protein (Ferguson *et al.,* 2017), and identifies a new role for AMS during late meiosis. This indicates that AMS has a dual function, with a previously unreported role during early pollen development in controlling late meiotic progression, as well as its subsequent role as a master regulator of pollen wall biosynthesis and formation. Despite gametophytic control of initiation of PMC meiosis, there is clear maternal regulation via the tapetum. This work identifies AMS as a key player in the crosstalk between the gametophyte and sporophytic tissues, which is essential to enable synchronous development of tapetum and microspores for functional pollen formation.

## Supplementary data

The following supplementary data are available at *JXB* online.

Fig. S1. Transverse sections through wild-type Col-0 and *ams* mutant anthers.

Fig. S2. 4ʹ,6-Diamidino-2-phenylindole (DAPI) staining of male sterile *ams* meiocytes during male meiosis showing formation of unbalanced tetrads.

Fig. S3. Callose staining of male meiocytes of tapetum defective mutants.

Fig. S4. Arabidopsis bud size in *ams* mutant in comparison with wild-type (Col-0) at different pollen stages.

Fig. S5. qRT-PCR analysis of key genes in tapetal cells that have been subject to fluorescence-activated cell sorting based upon expression of the tapetum-expressed A9–GFP fluorescent protein; and *in situ* hybridization analysis of *ML1* expression within sectioned wild-type anthers.

Table S1. Primers used in this study.

Table S2. Average expression stability of reference genes.

erac225_suppl_Supplementary_Materials

## Data Availability

All data supporting the findings of this study are available within the paper and within its supplementary materials published online.
