# Supplementary material for: Sporophytic control of pollen meiotic progression is mediated by tapetum expression of ABORTED MICROSPORES
Source: J Exp Bot. 2022 May 25;73(16):5543–58. doi: 10.1093/jxb/erac225 (PMC9467646; doi:10.1093/jxb/erac225)
Supplement: erac225_suppl_Supplementary_Materials [file erac225_suppl_supplementary_materials.pdf]

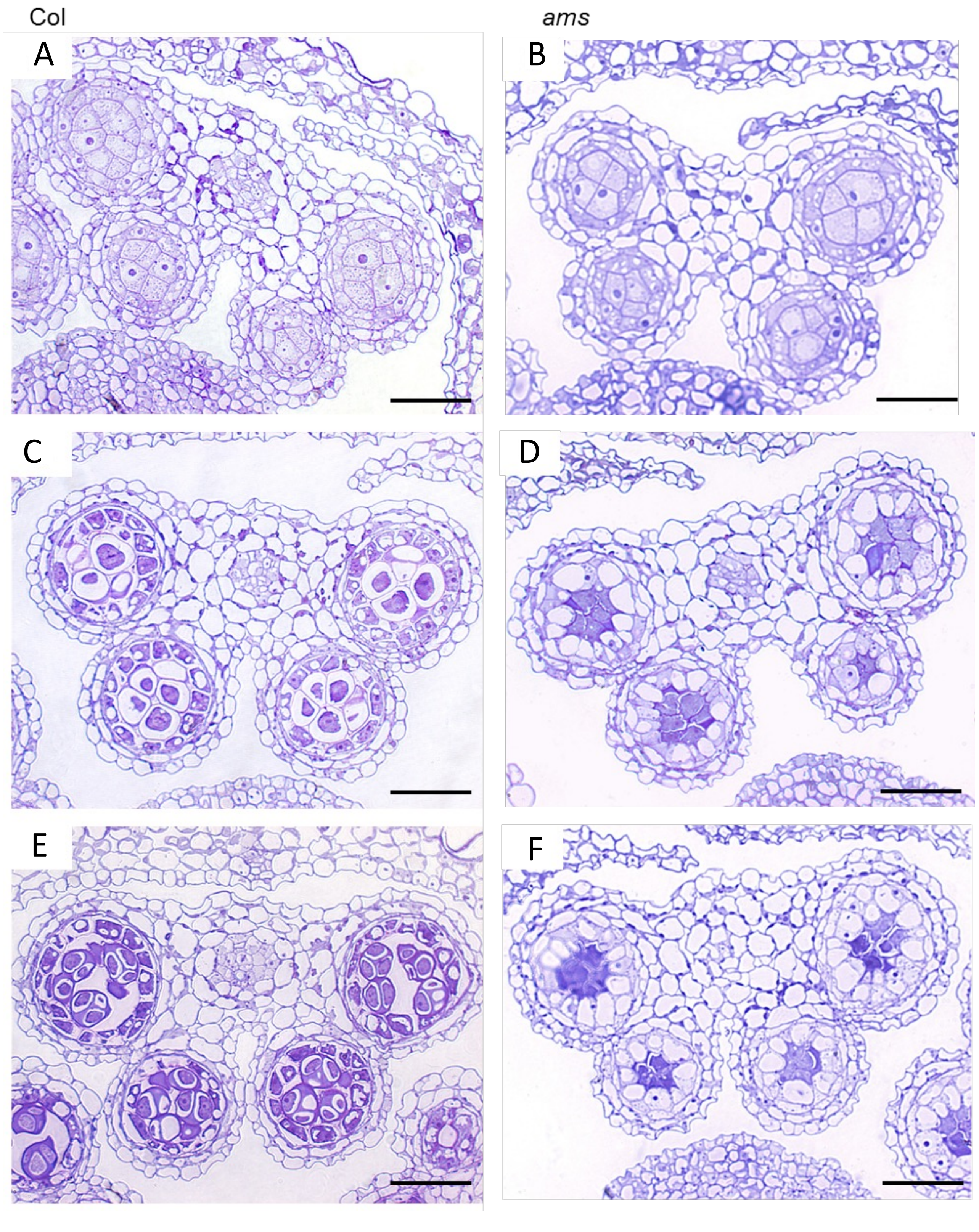

Supplementary Data Figure S1

Transverse sections through wild-type Col-0 (**A, C, E**) and *ams* mutant anthers (**B, D, F**).

(**A, B**) Pre-meiosis, (**C, D**) Meiosis, callose wall deposition, (**E, F**) Late Tetrad stage. Scale bars = 25 μm.

ams

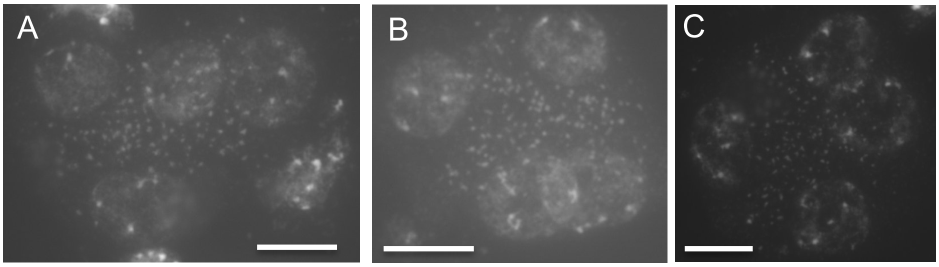

D

	Balanced tetrads (no.)	Percentage (%)	Unbalanced tetrads (no.)	Percentage (%)
Col-0	13	92.86	1	7.14
dyt1	2	40	3	60
tdf1	2	66.67	1	33.33
ams	17	73.91	6	26.09
ms1	3	100	0	0
ms188	10	100	0	0

Supplementary Data Figure S2

A-C) 4',6-Diamidino-2-phenylindole (DAPI) staining of male sterile mutants *ams* meiocytes during male meiosis showing formation of unbalanced tetrads. Scale bar = 10µm. **D)** table showing number and percentage of balanced and unbalanced tetrads observed.

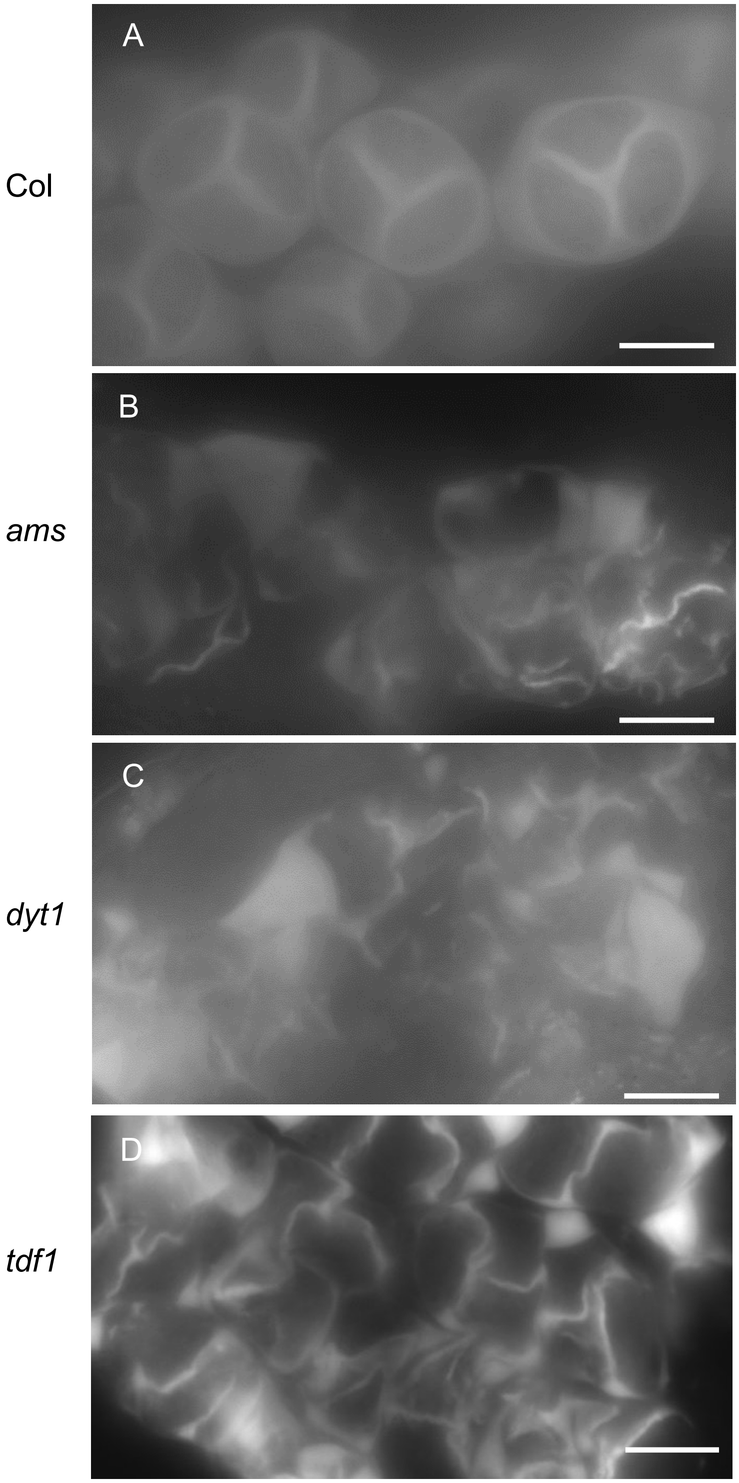

Supplementary Data Figure S3

Callose staining of male meiocytes of tapetum defective mutants. Callose staining of tetrads of wild-type Col (**A**), *ams* (**B**), *dyt1* (**C**) and *tdf1* (**D**) mutant plants, suggesting callose deposition and callosic cell wall organisation defects in mutants, with tetrads in mutants adjacent to each other (B, C, D). Scale bars = 10 µm.

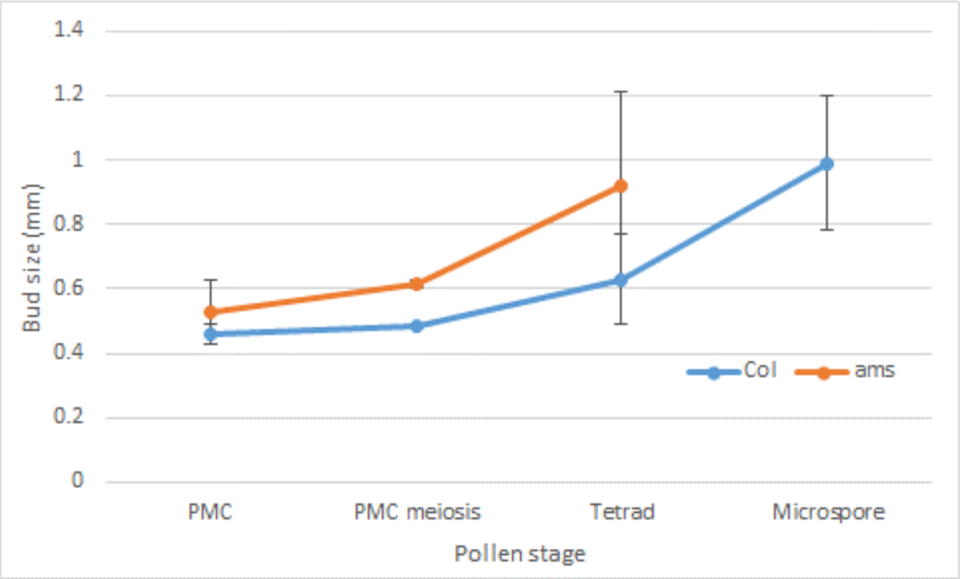

Supplementary Data Figure S4

Arabidopsis bud size in *ams* mutant in comparison to wild-type (Col-0) at different pollen stages, error bars show range of bud sizes that each stage is found at, based on DAPI staining. These results represent data collected from at least three biological replicates.

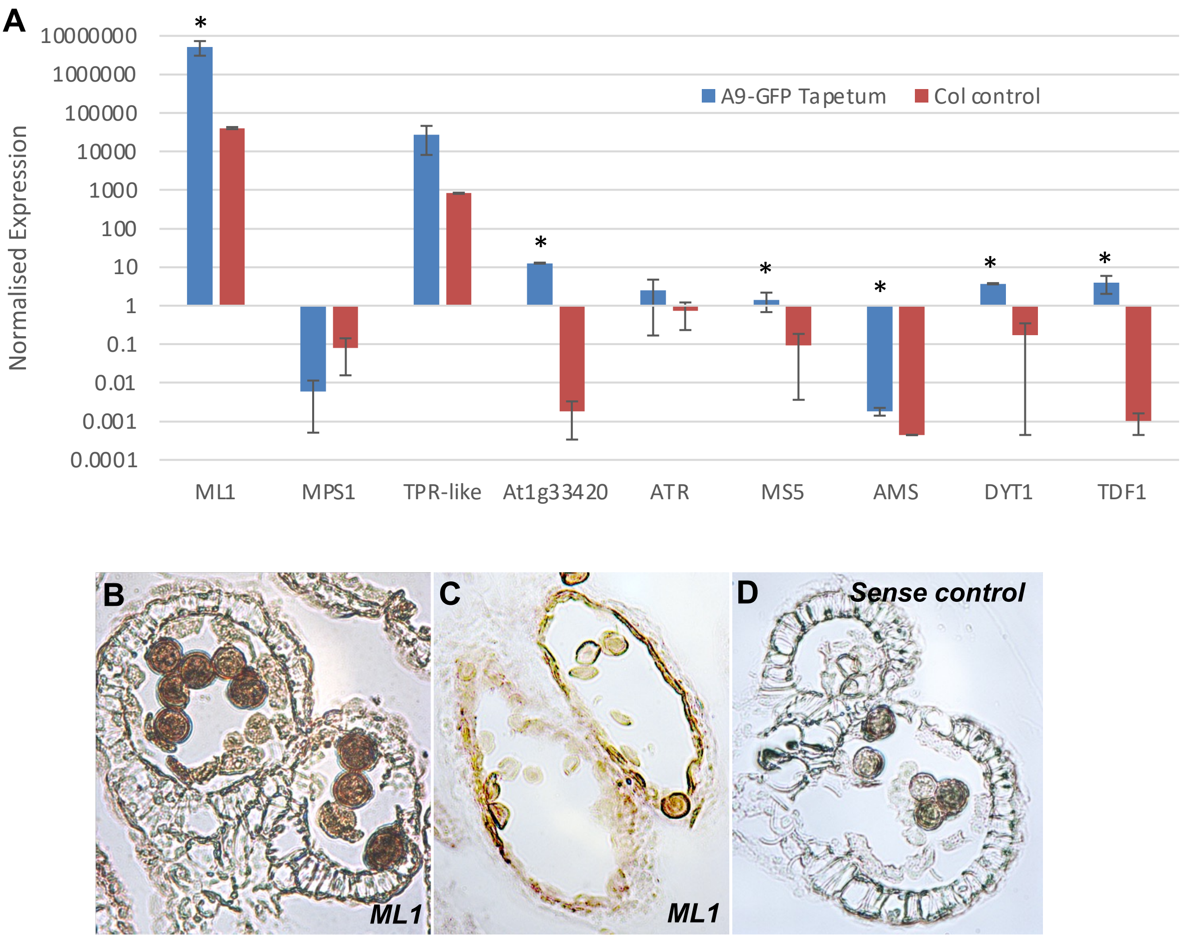

Supplementary Data Figure S5

A) qRT-PCR analysis of key genes in tapetal cells that have been subject to fluorescence-activated cell sorting based upon expression of the tapetum-expressed A9–GFP fluorescent protein; Col: FACS sorted cells showing background from experiment as no cells should be pulled down; A9-GFP tapetum: FACS sorted cells enriched for tapetum cells expressing the A9-GFP fusion protein. Error bars = standard deviation. These results represent data collected from at least three biological replicates. Significant changes based on students t-test P >0.05 are represented by *.

B) *In situ* hybridisation analysis of *ML1* expression within sectioned wild-type anthers using anti-sense (B, C) and sense control (D). Signal observed in tapetal cells and pollen, however signal also observed in the pollen in the control.
